# Aberrant ADAM10 expression correlates with osteosarcoma progression

**DOI:** 10.1186/2047-783X-19-9

**Published:** 2014-02-18

**Authors:** Ren Zhao, Dongjing Ni, Yi Tian, Bing Ni, Aimin Wang

**Affiliations:** 1 Department of Orthopedics, Daping Hospital, Third Military Medical University, No. 10 Changjiang Road, Daping, Yuzhong District, Chongqing 400042, China; 2 Institute of Immunology of PLA, Third Military Medical University, No. 30 Gaotanyan Street, Shapingba District, Chongqing 400038, China

**Keywords:** ADAM10, CD31, Multinucleated giant cell, Notch1, Osteoclast, Osteosarcoma, Tumor progression

## Abstract

**Background:**

Osteosarcoma is the most common type of bone cancer and is notorious for its rapid progression. The Notch signaling pathway has recently been shown to be involved in osteosarcoma. As a major sheddase of Notch receptors, ADAM10 has been implicated in many types of cancers, but its role in osteosarcoma has not been investigated. Previous studies have shown that the expression of CD31 was significantly elevated in metastatic osteosarcoma; however, its expression in nonmetastatic groups is not known. In addition, the mysterious multinucleated giant cell in giant cell-rich osteosarcoma was previously regarded as an osteoclast-like cell, but its exact identity is unclear.

**Method:**

Tissue chip samples from 40 cases of nonmetastatic osteosarcoma were stained for cytoplasmic ADAM10, activated Notch1 and CD31. Osteoclasts in tumor sections were also stained for tartrate-resistant acid phosphatase (TRAP).

**Results:**

Immunofluorescence staining revealed that ADAM10 expression significantly increased with the progression of osteosarcoma as well as in osteoblastic osteosarcoma, whereas the expression of the Notch intracellular domain (NICD) and CD31 was not significantly altered between different pathological stages. In addition, multinucleated giant cells in giant cell-rich osteosarcoma were also found to coexpress CD31, ADAM10 and NICD, but were negative for TRAP staining.

**Conclusions:**

Our results highlight the importance of ADAM10 in the progression of osteosarcoma and suggest that the protein might be a potential therapeutic target in osteosarcoma treatment. This study also demonstrates that the multinucleated giant cell is an angiogenic tumor cell, rather than an osteoclast, and involves ADAM10/Notch1 signaling activation.

## Background

Osteosarcoma, the most common type of primary bone cancer, is a malignant neoplasm notorious for its high aggressiveness and early metastatic potential [1]. However, little is known about the signaling pathways that are crucial for its progression, and the molecular biology of osteosarcoma remains poorly understood. Histologically, osteosarcoma is categorized into several different types, including osteoblastic osteosarcoma, chondroblastic osteosarcoma, fibroblastic osteosarcoma and giant cell-rich osteosarcoma. The giant cell-rich osteosarcoma is the rare subtype of primary osteogenic sarcoma, and, to the best of our knowledge, only 18 cases have been reported in the literature to date [2]. Its name derives from the existence of multinucleated giant cells within the tumor tissues, which were generally believed to be osteoclast-like tumor cells [2]; yet their exact identity still remains to be elucidated.

A class of disintegrins and metallproteinases, known as ADAMs, have been shown to participate in a variety of signaling events that are aberrant in cancers as well as during tumor progression [3]. ADAM10, a member of the ADAM family, has been found to be upregulated in many cancers, including ovarian, colon and prostate cancers [4,5]. Overexpression of ADAM10 promotes the growth of oral squamous cell carcinoma and gastric carcinomas, whereas downregulation of its expression reduces proliferation of carcinoma cells [6,7]. However, the role of ADAM10 in osteosarcoma is still unclear.

As a membrane-bound sheddase, ADAM10 cleaves a variety of cell surface proteins, including Notch receptors [8,9]. In the canonical Notch signaling pathway, Notch receptors interact with membrane-anchored ligands, followed by sequential proteolytic cleavage by ADAM10 and presenilin within the transmembrane, thus releasing the Notch intracellular domain (NICD), which enters the nucleus and regulates a cohort of Notch-dependent genes. Although aberrant Notch receptor expression has been reported in osteosarcoma [10] and manipulation of the Notch pathway has been shown to play a crucial role for *Hes1* (a Notch-dependent gene) in osteosarcoma invasion and metastasis [11,12], whether the ADAM10–Notch1 signaling axis is particularly involved in osteosarcoma is unknown.

CD31 is a member of the immunoglobulin (Ig) gene superfamily and plays an important role in a number of endothelial cell functions, including angiogenesis, inflammation, integrin activation and intercellular adhesion [13-15], which could also be employed by tumor cells for their progression. Indeed, on the basis of their immunohistochemical staining of primary and metastatic osteosarcoma samples, Arihiro *et al*. suggested that the formation of metastatic foci of osteosarcoma cells in other bones might be regulated by CD31 to promote endothelial cell migration [16]. Nevertheless, the role of CD31 in nonmetastatic osteosarcoma has not yet been revealed.

The goal of our present study was to investigate the expression of ADAM10, Notch1, CD31 and tartrate-resistant acid phosphatase (TRAP) in nonmetastatic osteosarcoma tissue and their respective contributions to tumor progression.

## Methods

### Tissue chips

Paraformaldehyde-fixed, paraffin-embedded human osteosarcoma tissue chip slides were purchased from US Biomax (Rockville, MD, USA). Each slide contained 40 duplicate samples of osteosarcoma tissues, and pathological stages included IA (*n* = 3), IB (*n* = 5), IIA (*n* = 10) and IIB (*n* = 22), according to the Musculoskeletal Tumor Society Staging System. This study was approved by the ethics committee of Daping Hospital of the Third Military Medical University.

### Reagents and antibodies

Mouse anti-human CD31 monoclonal antibody (Ab), rabbit anti-human ADAM10 (cytoplasmic domain) polyclonal Ab and rabbit anti-human activated Notch1 NICD polyclonal Ab were all purchased from Abcam (Cambridge, UK). Isotype-specific control Abs were also obtained from Abcam and were used to control staining specificity. Fluorescein isothiocyanate (FITC)-conjugated goat anti-mouse IgG Ab (ZSGB-BIO, Beijing, China) and cyanine 3 (Cy3)-conjugated goat anti-rabbit IgG Ab (Beyotime Institute of Biotechnology, Jiangsu, China) were used as secondary Abs. Antifade mounting medium was also obtained from Beyotime Institute of Biotechnology.

### Immunohistological and immunofluorescent staining

Paraffin tissue sections were deparaffinized in xylene, rehydrated through graded ethanol series and washed in 10 mM phosphate-buffered saline (PBS), pH 7.4. Hematoxylin and eosin (H&E) staining was performed according to a standard protocol. The histological types were assessed by an experienced pathologist specializing in osteosarcoma diagnosis. For immunofluorescent staining, antigen retrieval was performed by incubating tissue sections for 20 minutes in 0.01 M sodium citrate buffer, pH 6.0, and heated in a microwave oven, then sections were permeabilized with 0.1% Triton X-100. After blocking with 5% bovine serum albumin/PBS for 30 minutes, three sequential slides were then incubated with anti-CD31 Ab, anti-ADAM10 (cytoplasmic domain) Ab and anti activated Notch1 (NICD) Ab, respectively. For double-immunofluorescence staining, two additional sequential slides were incubated with either anti-CD31 Ab (mouse anti-human) plus anti-ADAM10 Ab (rabbit anti-human) or anti-CD31 Ab plus anti-NICD Ab (rabbit anti-human), respectively. Isotype-specific control Abs were used to test staining specificity. Slides were incubated overnight at 4°C. After being washed with PBS, slides were stained with secondary FITC anti-mouse Ab and Cy3 anti-rabbit Ab for 30 minutes at room temperature (RT). Afterward, nuclei were stained with 4′,6-diamidino-2-phenylindole (DAPI) for 10 minutes at RT. Images were obtained using the MRC-600 digital confocal laser scanning system (Bio-Rad Laboratories, Hercules, CA, USA).

### TRAP/DAPI staining

TRAP staining was performed on deparaffinized and rehydrated sections using the Acid Phosphatase, Leukocyte (TRAP) Kit (Sigma-Aldrich, St Louis, MO, USA) according to the manufacturer’s instructions. DAPI staining was performed afterward as mentioned above to visualize the nuclei.

### Data analysis

Acquired images were exported and merged using Image-Pro Plus version 6.0 software (MediaCybernetics, Rockville, MD, USA). NICD and CD31 density percentages were measured as the percentage of positive FITC signal area against the whole field area under 100× magnification as described by Connor *et al*. [17]. ADAM10-positive tumor cells were counted in ADAM10/DAPI merged images under the same magnification, and analysis was performed with tools in Image-Pro Plus software. Statistical differences were determined using a two-tailed, unpaired Student’s *t*-test.

## Results

### ADAM10 expression was significantly increased in more advanced and osteoblastic osteosarcoma

**Figure 1:**
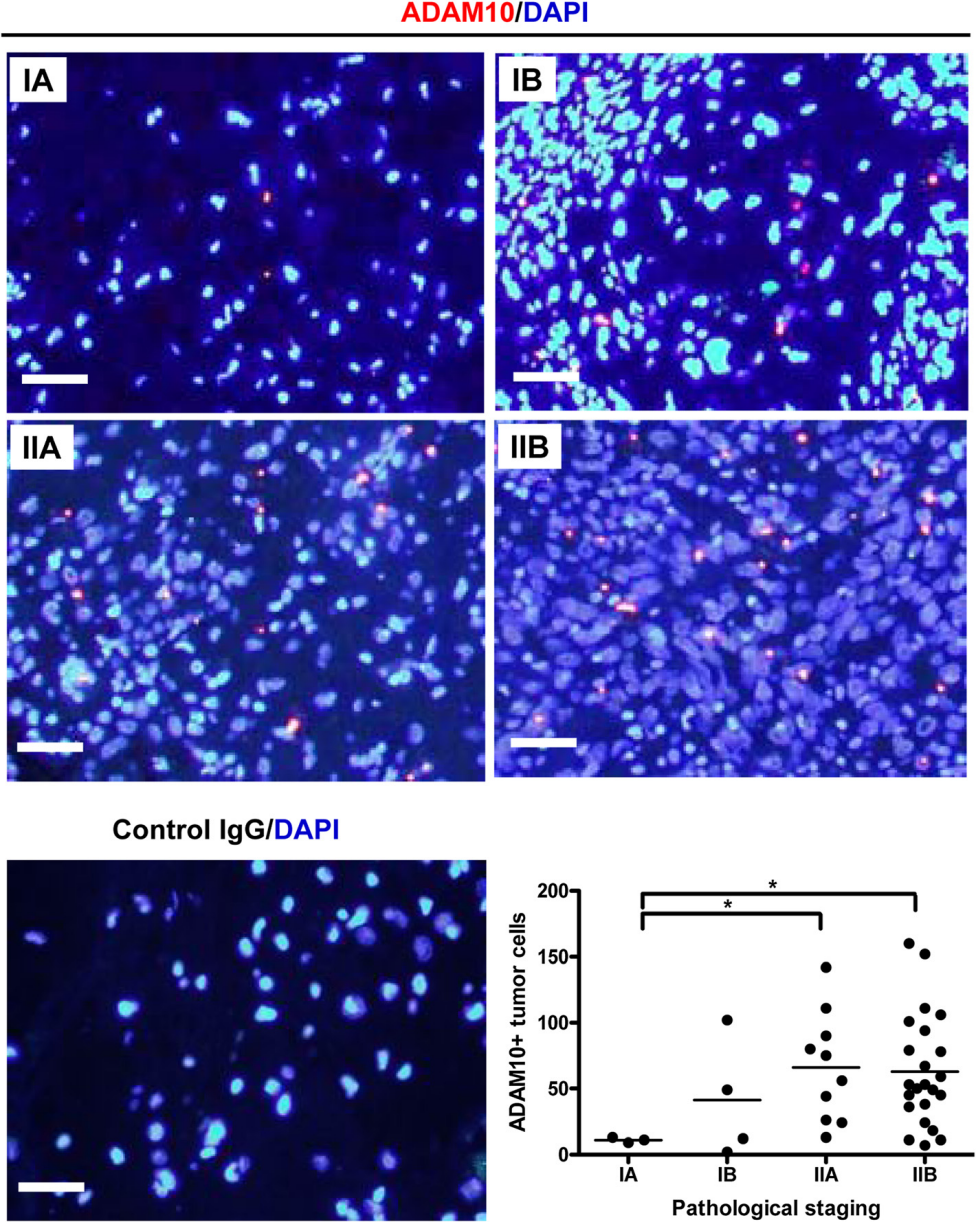
**Increased expression of ADAM10 in higher**-**stage osteosarcoma tissue.** The panels show representative images of the cytoplasmic ADAM10 domain (red dots) merged with 4′,6-diamidino-2-phenylindole (DAPI)-stained nuclei (blue) taken from osteosarcoma tissues pathologically staged from IA to IIB. Specific ADAM10 staining was controlled by using isotype-specific control antibody (control immunoglobulin G (IgG)). The lower-right graph shows statistical analysis of the number of ADAM10^+^ tumor cells in osteosarcoma tissues at different stages. ADAM10: A disintegrin and metalloproteinase 10. Scale bar, 20 μm. *P < 0.05.

We first examined the expression of ADAM10 in osteosarcoma tissue chips. Under 400× magnification, a polarized and condensed expression pattern of cytoplasmic ADAM10 was observed in all cases and stages of osteosarcoma (Figure 1). In order to determine whether ADAM10 was differentially expressed among different stages of osteosarcoma, ADM10^+^ tumor cells were counted under 100× ADAM10/DAPI merged images. The number of ADM10^+^ tumor cells in stages IIA and IIB increased significantly compared with those in stage IA (Figure 1), suggesting that elevated ADAM10 levels in tumor cells might be involved in the progression of osteosarcoma.

After the immunofluorescent staining, osteosarcoma tissue chips were stained with H&E. Four types of osteosarcoma were recognized, including giant cell-rich osteosarcoma, osteoblastic osteosarcoma, fibroblastic osteosarcoma and chondroblastic osteosarcoma (Additional file 1: Figure S1, upper panels). We then reanalyzed the number of ADAM10^+^ tumor cells based on different histological types and found that osteoblastic osteosarcoma had significantly more ADAM10^+^ tumor cells compared with chondroblastic and fibroblastic osteosarcomas (Additional file 1: Figure S1, lower graph), suggesting that ADAM10 might be involved specifically in the pathogenesis of osteoblastic osteosarcoma.

### Activated Notch1 was not markedly altered in different stages of osteosarcoma

**Figure 2:**
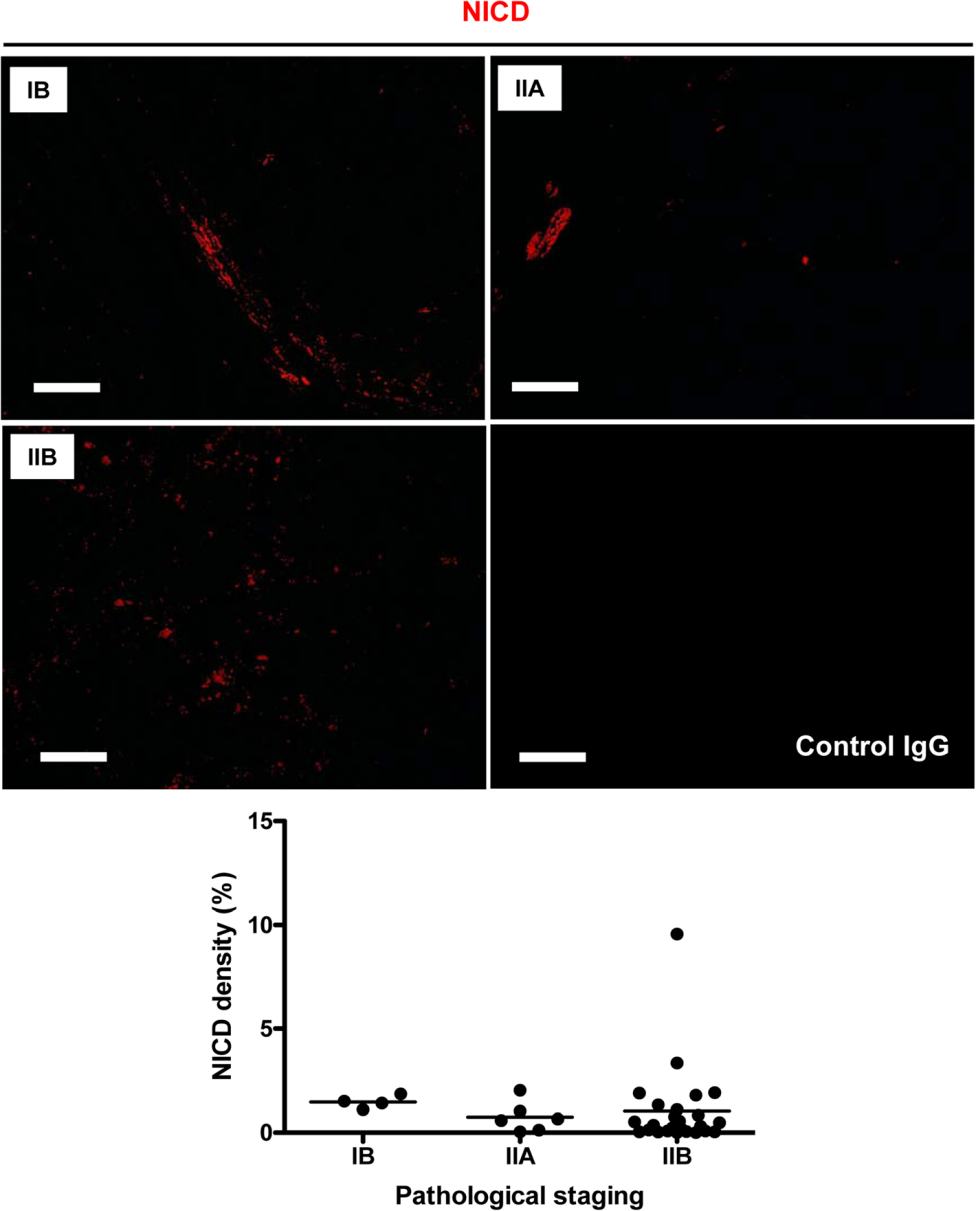
**Expression of activated Notch1 in different stages of osteosarcoma.** The panels show representative images of Notch intracellular domain (NICD; red) expression in stages IB, IIA and IIB osteosarcoma tissues. Specific NICD staining was controlled by using isotype-specific control (control immunoglobulin G (IgG)) antibody. The lower graph shows the statistical analysis of the density of NICD^+^ cells in osteosarcoma tissues at different stages. Scale bar, 80 μm.

As ADAM10 is a major regulator of Notch signaling via its shedding of Notch receptors [8,9], we sought to determine whether increased expression of ADAM10 in osteosarcoma progression would also lead to increased activation of Notch1. We detected the expression of activated Notch1 (NICD) in another sequential slide. As shown in the upper panels of Figure 2, Notch1 was activated in osteosarcoma tissue at all stages; however, its activation was not significantly correlated with pathological staging, as shown by NICD density from stage IB to stage IIB. These results suggest that Notch1 was activated in osteosarcoma but that its activation might not contribute to disease progression.

### CD31 might not contribute to osteosarcoma progression

**Figure 3:**
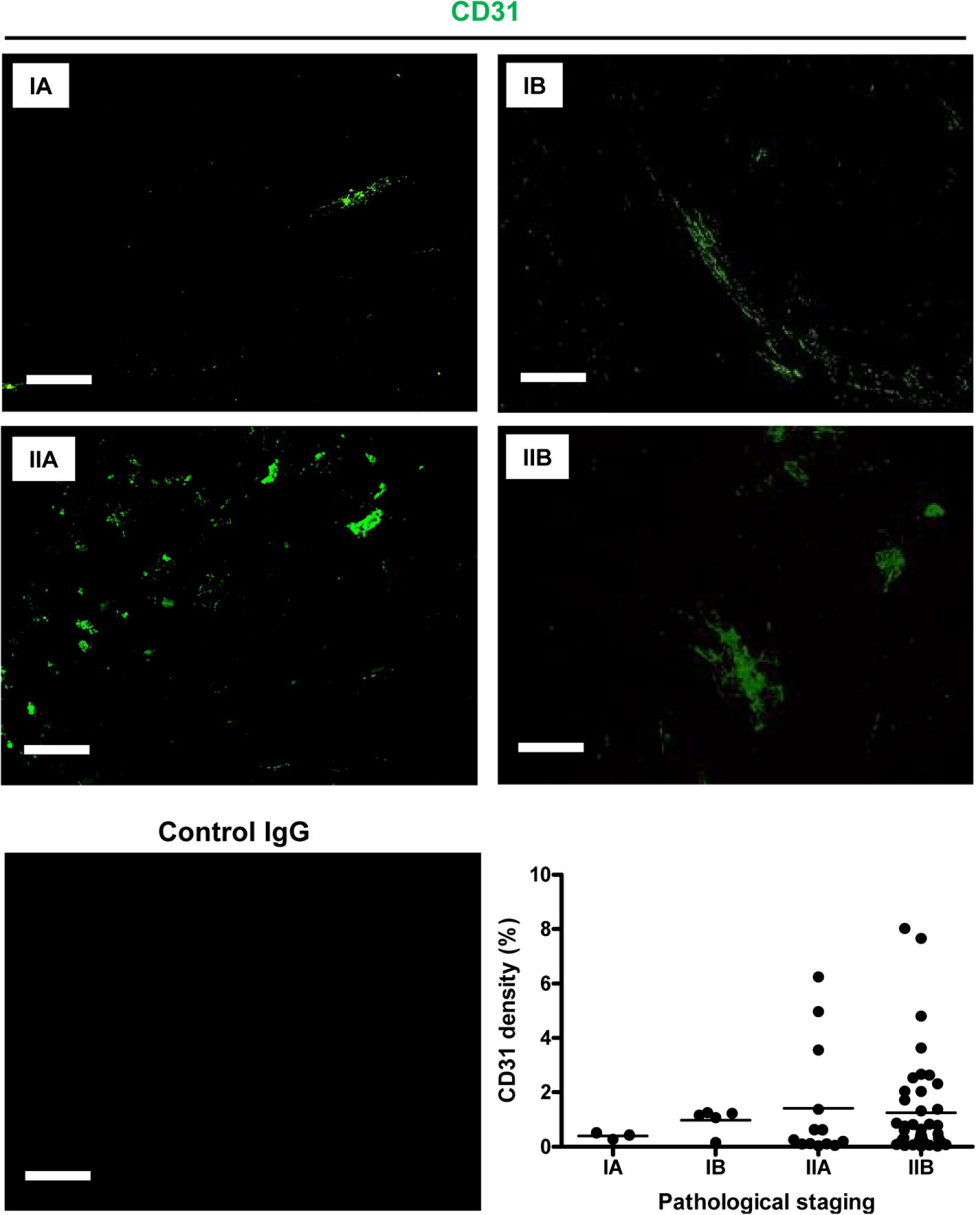
**Expression of CD31 in different stages of osteosarcoma.** The panels show representative images of CD31 expression (green) from stages IA to IIB osteosarcoma tissues. Isotype-specific control antibody (control immunoglobulin G (IgG)) was used to show the specificity of CD31 staining. The lower-right graph shows statistical analysis of CD31density in osteosarcoma tissues at different stages. Scale bar, 80 μm.

Previous studies have shown that the expression of CD31 was significantly increased in metastatic osteosarcoma compared with primary osteosarcoma [16]. However, the differences between nonmetastatic groups are unclear. Hence we performed FITC-CD31 staining on all osteosarcoma tissues from stages IA to IIB. Although a trend of increased CD31 expression was observed as osteosarcoma progressed, no significant differences were found between different pathological stages in terms of CD31 density (Figure 3), suggesting that vascularity (represented by the expression of CD31) was not dramatically altered during the pathological progression of nonmetastatic osteosarcoma, a scenario different from metastatic groups.

### Multinucleated giant cell is angiogenic tumor cell, not osteoclast

**Figure 4:**
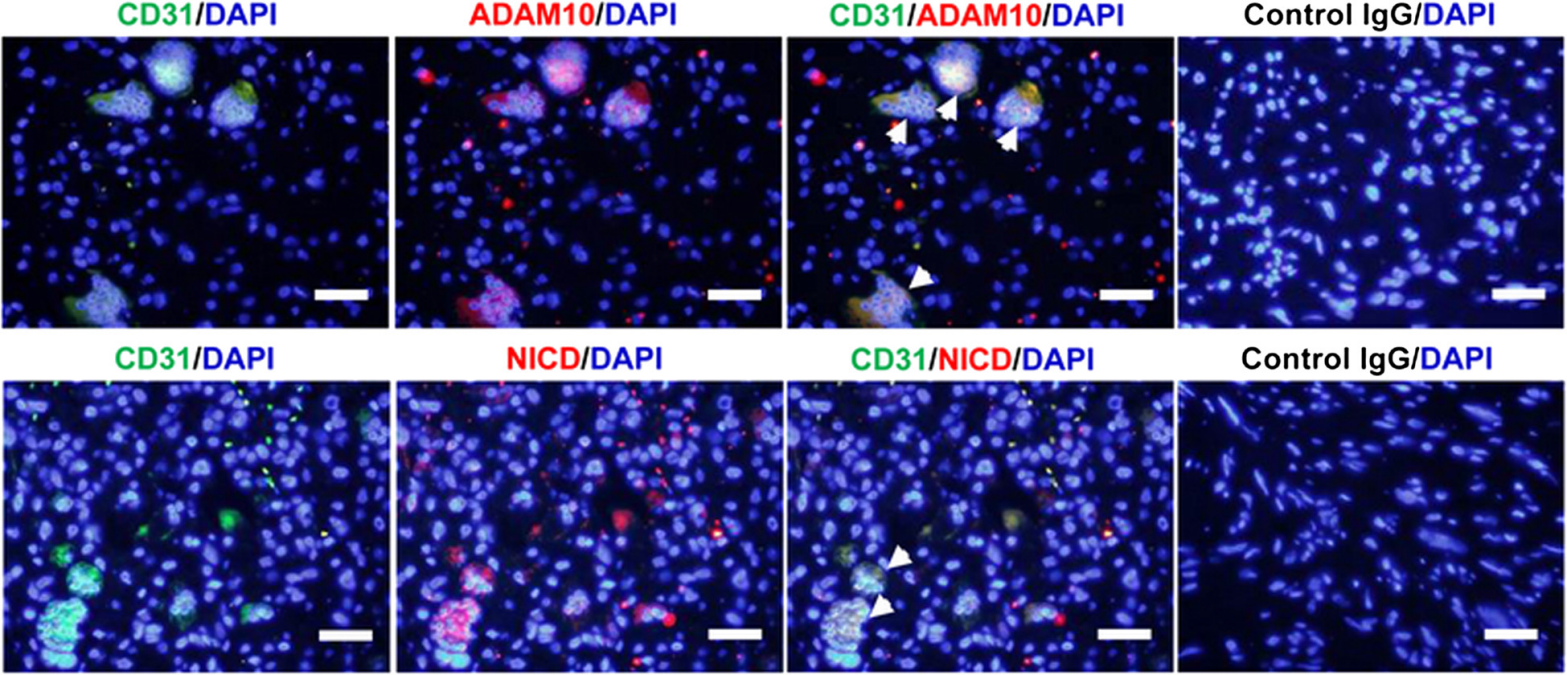
**Expression of CD31, cytoplasmic ADAM10 and activated Notch1 in multinucleated giant cells.** In giant cell-rich osteosarcoma sections, CD31 expression was visualized with mouse anti-human monoclonal antibody (Ab), followed by fluorescein isothiocyanate (FITC)-coupled goat anti-mouse secondary Ab (green). ADAM10 and Notch intracellular domain (NICD) expression were detected by rabbit anti-human polyclonal Abs, respectively, and cyanine 3-coupled goat anti-rabbit secondary Ab (red). Nuclei of osteosarcoma cells were stained with 4′,6-diamidino-2-phenylindole (DAPI). Arrowheads in the merged pictures indicate colocalization of CD31/ADAM10 and CD31/NICD in multinucleated osteosarcoma tumor cells. Isotype-specific control immunoglobulin G (IgG) Abs were used to test staining specificity. ADAM10: A disintegrin and metalloproteinase 10. Scale bar, 20 μm.

**Figure 5:**
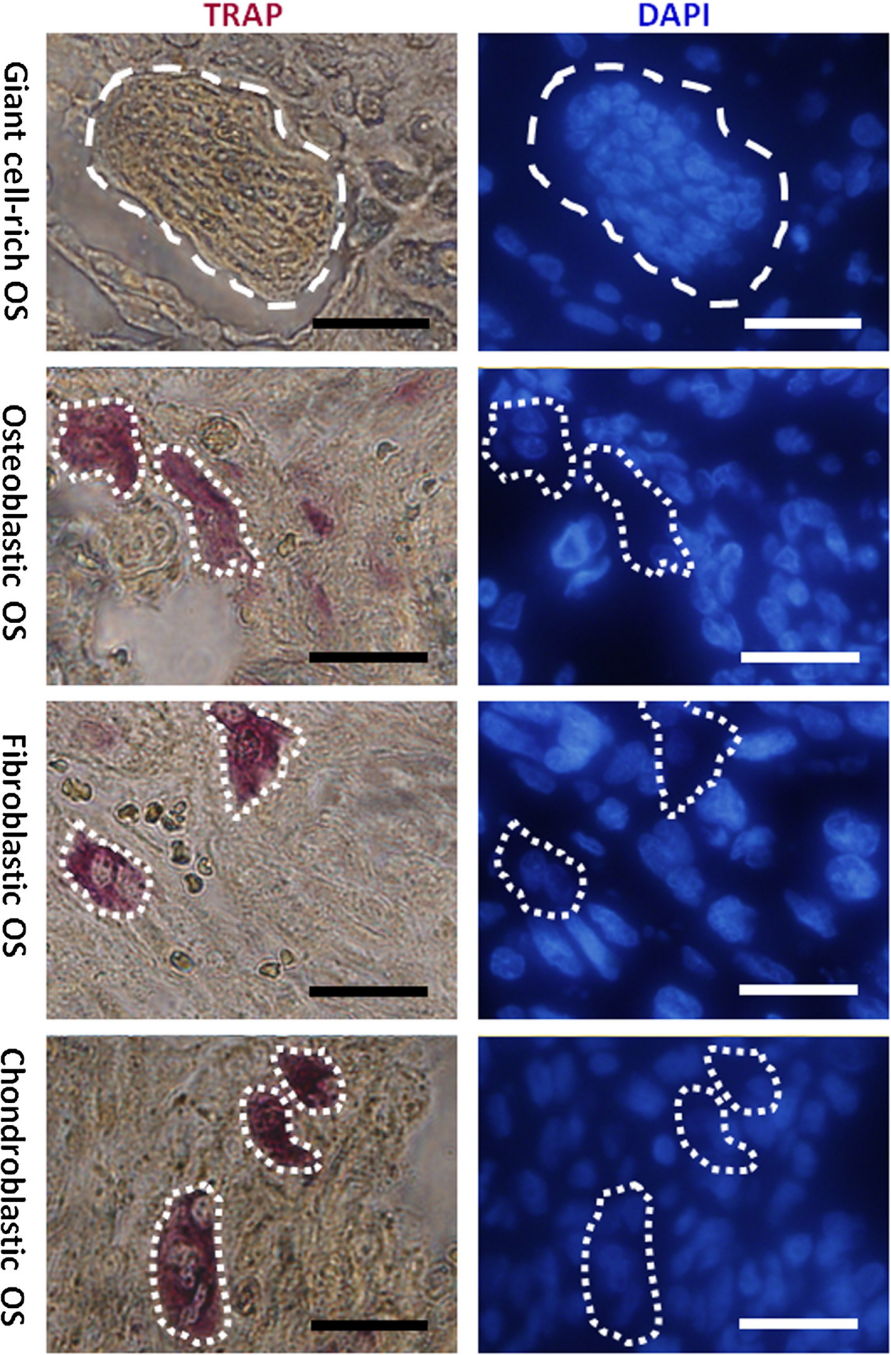
**TRAP/DAPI staining of various histological types of osteosarcoma.** The left panels show tartrate-resistant acid phosphatase (TRAP) staining of giant cell-rich, osteoblastic, fibroblastic and chondroblastic osteosarcomas, respectively. The right panels show 4′,6-diamidino-2-phenylindole (DAPI) staining of the nuclei of sections identical to those on the left panels. Note that negative TRAP staining was observed in multinucleated giant cells (upper panel). Dashed lines outline the cell contours. Scale bar, 20 μm.

We found that the multinucleated giant cells in giant cell-rich osteosarcoma stained positively with CD31 (Figure 4, left panels), suggesting their angiogenic property. Moreover, cytoplasmic ADAM10 and activated Notch1 were colocalized within these giant cells (Figure 4), suggesting that ADAM10/Notch1 signaling was activated in these angiogenic tumor cells. By TRAP staining, we observed that TRAP was absent in multinucleated cells in giant cell-rich osteosarcoma tissue, whereas TRAP^+^ cells were found in osteoblastic, fibroblastic and chondroblastic osteosarcoma tissue (Figure 5). Taken together, these results suggest that what were previously called osteoclast-like multinucleated giant cells [2] are not osteoclasts, but rather angiogenic tumor cells that involve ADAM10/Notch1 signaling activation.

## Discussion

Although ADAM10 has been found to be upregulated in many cancers [4,5], its expression in tumors with mesenchymal origin (particularly osteosarcoma) remains unknown. Herein we show that ADAM10 was generally expressed in osteosarcoma tumor cells in a condensed and polarized pattern. The level of ADAM10 in tumor cells significantly increases as osteosarcoma progresses, as well as in osteoblastic osteosarcoma tissue, suggesting that it might play a role in osteosarcoma tumor progression and the pathological development of osteoblastic osteosarcoma. As ADAM10 is a major sheddase of Notch receptors [8,9], and because researchers in recent studies have discovered that Notch signaling was activated in human osteosarcoma and may play a role in tumor invasion and metastasis [11,18,19], we speculate that ADAM10 might participate in osteosarcoma progression through the shedding of Notch receptors and activation of the Notch signaling pathway.

Currently, there is no systematic evaluation comparing Notch expression in clinical osteosarcoma between different pathological stages. Hence, we analyzed activated Notch1 (NICD) expression from stages IB to IIB at the protein level and found no significant alteration in NICD density. Therefore, as one of four members in the mammalian Notch receptor family, Notch1 might not be the Notch receptor responsible for osteosarcoma progression. Similarly, Tanaka *et al*. found that expression of Notch1 mRNA was decreased in specimens from human osteosarcoma biopsies compared with normal bone tissues [20].

Previous studies have shown that CD31 expression was significantly correlated between primary and metastatic osteosarcomas [16], but the correlation between nonmetastatic groups is not clear. In our present study, we found that variation in the density of CD31 was not significantly associated with osteosarcoma staging from IA to IIB, although there was a trend toward increased CD31 expression as osteosarcoma progressed.

The expression of CD31 and activation of Notch1 did not dramatically contribute to osteosarcoma progression overall. In the specific case of multinucleated tumor cells in giant cell-rich osteosarcoma, however, the expression of CD31 may facilitate the adhesion of these tumor cells to endothelial cells. Migration between endothelial cells may also occur via homophilic interactions between CD31 and CD31 and heterophilic interactions between CD31 and α_v_β_3_ integrin, which has been shown to be expressed in both osteosarcoma cells and endothelial cells [14,21,22], thus promoting tumor cell migration. Given the fact that these tumor cells also coexpress ADAM10 and activated Notch1, ADAM10/Notch1 signaling might also participate in this process, as recent studies have demonstrated that ADAM10 and Notch1 could promote the migration of a variety of tumor cells, both cooperatively [23] and individually [24,25]. Furthermore, the absent staining of TRAP and positive staining of CD31 in multinucleated giant cells suggest that these cells are not osteoclasts, but rather a type of angiogenic tumor cells in which ADAM10/Notch1 signaling is activated.

## Conclusion

In our present study, we examined the expression of CD31, ADAM10, Notch1 and TRAP in nonmetastatic osteosarcoma tissue chips. We found that only ADAM10 expression was significantly correlated with tumor progression and osteoblastic osteosarcoma development. Our results also suggest that multinucleated giant cells are not osteoclasts, but angiogenic tumor cells in which ADAM10/Notch1 signaling is activated and might be implicated in tumor cell migration. The results of this study imply heterogeneity in osteosarcoma between metastatic and nonmetastatic types and complexity within nonmetastatic types. It also highlights ADAM10 as a potential target for the biological intervention of nonmetastatic osteosarcoma.

## Competing interests

The authors declare that they have no competing interests.

## Authors’ contributions

ZR designed the study, carried out the immunohistological and immunofluorescence studies and TRAP staining experiments, collected and analyzed the data and drafted the manuscript. NDJ participated in the immunofluorescence experiment. TY helped to analyze the data. NB and WAM participated in the study design and revised the manuscript. All authors read and approved the final manuscript.

## Supplementary Material

Additional file 1: Figure S1. Analysis of ADAM10^+^ cells in various histological types of osteosarcoma. The upper panels show hematoxylin and eosin staining of giant cell-rich, osteoblastic, fibroblastic, and chondroblastic osteosarcoma tissue, respectively. The lower graph shows the number of tumor cells expressing ADAM10 in chondroblastic, fibroblastic, and osteoblastic osteosarcoma tissue. Scale bar, 20 μm. NS, not significant *P < 0.05, ***P < 0.001. Click here for file
