# Supplementary figures and images for: Aberrant ADAM10 expression correlates with osteosarcoma progression
Source: Eur J Med Res. 2014 Feb 18;19(1):9. doi: 10.1186/2047-783X-19-9 (PMC3936952; doi:10.1186/2047-783X-19-9)

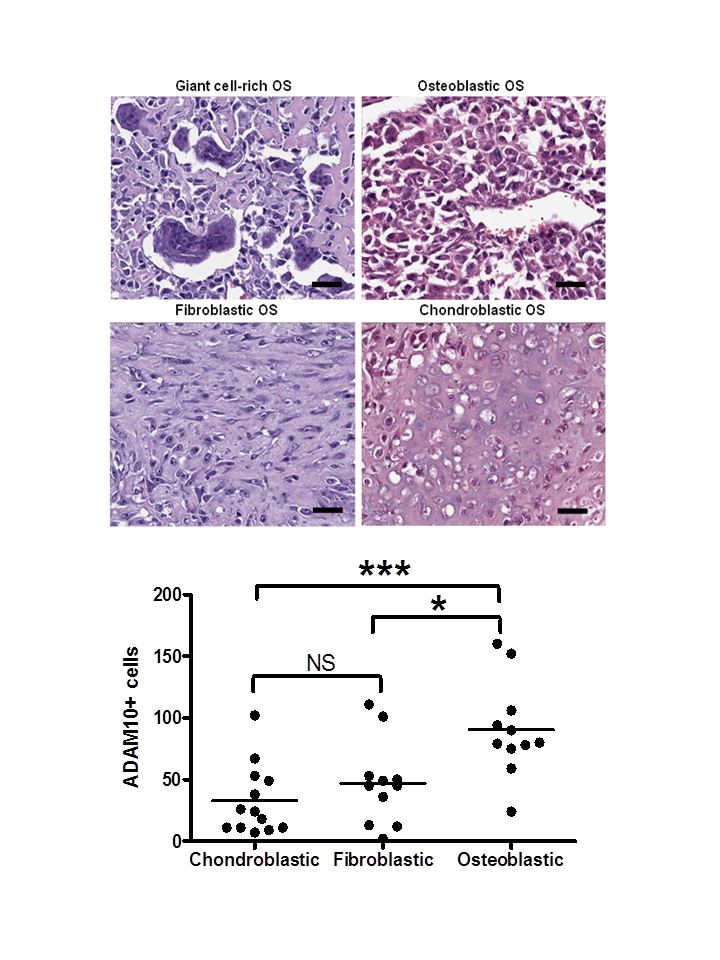

Supplement: Additional file 1: Figure S1. — Analysis of ADAM10+ cells in various histological types of osteosarcoma. The upper panels show hematoxylin and eosin staining of giant cell-rich, osteoblastic, fibroblastic, and chondroblastic osteosarcoma tissue, respectively. The lower graph shows the number of tumor cells expressing ADAM10 in chondroblastic, fibroblastic, and osteoblastic osteosarcoma tissue. Scale bar, 20 μm. NS, not significant *P < 0.05, ***P < 0.001. [file 2047-783X-19-9-S1.tiff]
